# Unveiling hydrogen chemical states in supersaturated amorphous alumina via machine learning-driven atomistic modeling

**DOI:** 10.1038/s41524-025-01676-5

**Published:** 2025-06-06

**Authors:** Simon Gramatte, Olivier Politano, Noel Jakse, Claudia Cancellieri, Ivo Utke, Lars P. H. Jeurgens, Vladyslav Turlo

**Affiliations:** 1 Laboratory for Advanced Materials Processing, Empa - Swiss Federal Laboratories for Materials Science and Technology, Feuerwerkerstrasse 39, Thun, CH-3602 Switzerland; 2 Laboratoire Interdisciplinaire Carnot de Bourgogne ICB UMR 6303, Université Bourgogne Europe, CNRS, Dijon, FR-21000 France; 3 Laboratory for Joining Technologies and Corrosion, Empa - Swiss Federal Laboratories for Materials Science and Technology, Ueberlandstrasse 129, Duebendorf, CH-8600 Switzerland; 4 National Centre for Computational Design and Discovery of Novel Materials (MARVEL), Empa - Swiss Federal Laboratories for Materials Science and Technology, Feuerwerkerstrasse 39, Thun, CH-3602 Switzerland; 5 Grenoble-INP, SIMaP, Université Grenoble Alpes, CNRS, Grenoble, FR-38000 France; 6 Laboratory for Mechanics of Materials and Nanostructures, Empa - Swiss Federal Laboratories for Materials Science and Technology, Feuerwerkerstrasse 39, Thun, CH-3602 Switzerland

**Keywords:** Chemistry, Materials science

## Abstract

Advancing hydrogen-based technologies requires detailed characterization of hydrogen chemical states in amorphous materials. As experimental probing of hydrogen is challenging, interpretation in amorphous systems demands accurate structural models. Guided by experiments on atomic layer deposited alumina, a fast atomistic simulation technique is introduced using an ab initio-based machine learning interatomic potential to generate amorphous structures with realistic hydrogen contents. As such, the annealing of highly defective crystalline hydroxide structures at atomic layer deposition temperatures reproduces experimental density and structure, enabling accurate prediction of Al Auger parameter chemical shifts. Our analysis shows that higher hydrogen content favors OH ligands, whereas lower hydrogen content leads to diverse chemical states and hydrogen bonding, consistent with charge density and partial Bader charge calculations. Our approach offers a robust route to link hydrogen content with experimentally accessible chemical shifts, aiding the design of next-generation hydrogen-related materials.

## Introduction

The energy transition of our society requires an improved fundamental understanding of the chemical interaction of H with different oxide materials^1^. For example, incorporation and diffusion of atomic H in oxide membranes for H-purification^2–4^, in oxides for photocatalytic water splitting^5,6^, as well as in passivated oxides on steel^7–9^, may affect the mechanical, chemical, and electronic properties and thereby the materials’ performance, reliability, and sustainability. In particular, the effect of H impurities on the barrier properties of oxide layers grown by Atomic Layer Deposition (ALD) is of great scientific and technological interest, since hydrogen permeation barriers fabricated by ALD are broadly applied to address specific challenges for transport, handling, and storage of H^1,10^. ALD also helps in the design of new advanced materials for energy conversion and storage^11–17^, electronics^18,19^, catalysis^20,21^, gas sensing^22^, biosensing^23^, water purification and gas separation^24,25^, as well as corrosion protection^26,27^.

**Fig. 1:**
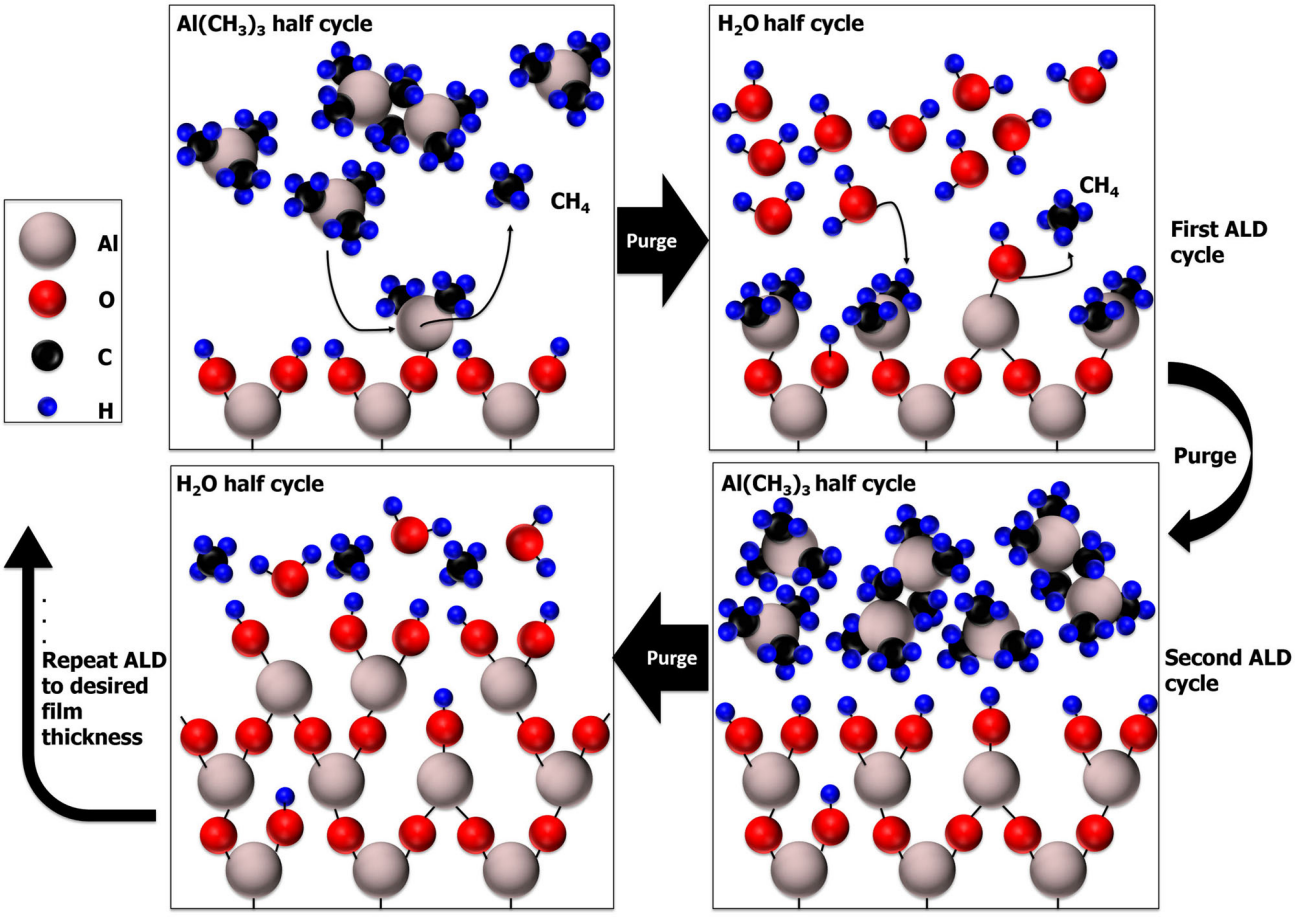
A schematic of the ALD process. A schematic of the ALD process for amorphous alumina using trimethylaluminum (TMA) and water precursors.

ALD represents a conventional method for creating functional thin films with remarkable atomic-level precision in thickness^28^, ensuring uniform, flawless coverage over complex substrate shapes, including those with high aspect ratios^29^ and porous structures^30,31^. Functioning as a specialized form of chemical vapor deposition, ALD operates through sequential exposure of substrates to vapor-phase precursors, which engage in self-limiting chemical reactions (known as chemisorption) on the substrate’s surface^32^, thus enabling the precise deposition of different material classes, such as metals^33,34^, simple binary oxides^35–37^, complex multicomponent compounds^38,39^, and 2D materials^40^. The ALD deposition process of barrier films of amorphous alumina includes alternating exposure of the activated surface to trimethylaluminum and water having methane as a reaction product. It was shown that a significant amount of hydrogen can be incorporated into these amorphous alumina ALD barrier films, especially at low growth temperatures, through unreacted OH ligands as highlighted in Fig. 1^41,42^. These H impurities have a detrimental impact on the mechanical properties^43,44^ while also lowering the dielectric strength^45^ and room-temperature thermal conductivity^46^ of the amorphous alumina films. The density and composition of the amorphous alumina ALD films in dependence on their hydrogen content (which is a function of the ALD growth temperature) was resolved experimentally^41,42^. However, to fundamentally rationalize the deterioration of the amorphous alumina film properties by H incorporation, it is critical to resolve hydrogen’s local chemical bonding state in the amorphous structure. It presents a huge challenge for modern experimental characterization tools due to their inability to probe hydrogen directly.

However, one chemical analysis technique such as X-ray photoelectron spectroscopy (XPS) stands out due to its ability to probe both the chemistry and structure of materials deep inside the thin films, which has the potential of indirectly probing hydrogen by analyzing its effects on other elements. At the cornerstone of such analysis is the determination of the so-called modified Auger parameter *α* representing the sum of the binding energy of core photoelectrons and the kinetic energy of core Auger electrons. Crucially, *α* remains inherently independent of the photon source, the chosen reference state (e.g. the Fermi level), and any charging effects during measurement, offering a robust and reliable metric for chemical state characterization. The special utility of Auger parameter analysis is its relation to extra-atomic relaxation energy *R*^ea^ that depends on the local atomic environment:

1 $$\Delta \alpha \approx 2{R}^{{\rm{ea}}}\approx \frac{14.4n\zeta }{D\zeta r+{r}^{4}},$$

where *Δα* is the Auger parameter shift (with respect to the gas-phase value (*α*_gas_)). The scaling factor 14.4 converts the energy into eV. Here, *n* represents the nearest-neighbor coordination number of the core-ionized atom, *ζ* is the electronic polarizability volume of the ligand field, and *r* is the distance from the core-ionized atom to a ligand. The parameter *D* is a geometrical factor that weights the contributions from individual ligands and is defined by

2 $$D=\sum _{j\ne i}\frac{1+{\cos }^{2}{\theta }_{ij}}{8{\cos }^{3}{\theta }_{ij}},$$

with *θ*_*ij*_ being the angle between the vectors connecting the randomly selected ligand *i* with the core-ionized atom (reference vector) and with another ligand *j*^47^. This geometrical parameter accounts for the anisotropic screening effects arising from the ligands’ spatial arrangement. By incorporating both the electronic polarizability and the detailed ligand geometry, the expression provides a comprehensive framework for understanding the modulation of the Auger parameter shift in complex systems. Equation (1) was originally developed for highly symmetric crystalline materials, where the parameters *n*, *D*, *ζ*, and *r* are assumed to be constant throughout the compound. We hypothesize that the model can be extended to some amorphous materials (should be determined in each particular case), provided that reliable structural averages, i.e., expected values of these parameters, can be estimated. Validating such a hypothesis for the Al-O-based systems is one of the key objectives of this work, as it would greatly simplify the analysis of general trends while moving from one structure to the other.

Therefore, in a first step, the atomic structure of such films should be accurately resolved with modern atomistic modeling tools, which is a very challenging problem to solve. First of all, significant uncertainty is present in the chemistry, structure, and properties of amorphous oxides produced by different fabrication methods: e.g. by thermal oxidation or anodization of pure metals^48–50^, by magnetron sputtering^51,52^, or by ALD^41,42^. In the latter, the incorporation of hydrogen further complicates the extraction of reliable experimental data for benchmarking atomistic simulations. Moreover, there is inherent uncertainty associated with the atomistic simulation procedures of amorphous structures, as state-of-the-art approaches for modeling amorphous alumina ALD films rely on melt-quenching, with insertion of hydrogen after melting and quenching of a crystalline oxide material^53^, or without considering H at all^54^. In the melt quenching method, the resulting structure will depend heavily on fabrication parameters such as the annealing temperature, the duration of the annealing, and the quenching rate^55^. Additional degrees of freedom include the choice of time step, thermostat, and barostat types and parameters, and most importantly the choice of the interatomic interaction model. Regarding the latter, even state-of-the-art machine learning interatomic potentials (MLIP) can be only as good as the underlying ab initio data used in their training^56^. For example, metal oxide systems like amorphous alumina in which nominal charge neutrality is not preserved (thus having charged defects) fall outside the purview of classical Density Functional Theory (DFT) calculations employing the Perdew–Burke–Ernzerhof (PBE) level of accuracy^57^ - the level commonly used for training machine learning models^56,58,59^.

**Fig. 2:**
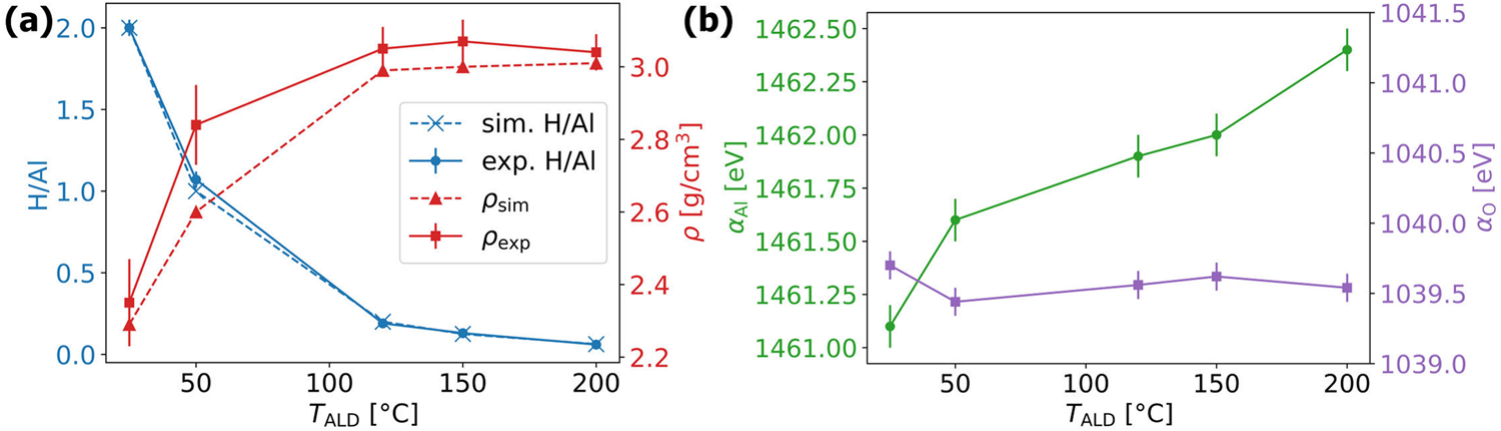
Summary of the experimental results. Summary of the experimental results from Cancellieri et al.^42^. **a** Dependence of the ALD process temperature, *T*_ALD_, on the stoichiometry, represented by the H/Al ratio (spheres), and the density, *ρ* (squares), of ALD alumina films. The left y-axis in blue indicates the H/Al ratio, while the right y-axis in red shows the density *ρ*. For comparison, the stoichiometry and densities of simulated ALD films are plotted as crosses and diamonds, respectively, and connected with dashed lines. **b** Variation of the Auger parameters measured by XPS/HAXPES for Al *α*_Al_ (spheres) and O *α*_O_ (squares) as a function of *T*_ALD_. The y-axis for *α*_Al_ is on the left and colored green, while that for *α*_O_ is on the right and colored purple.

In this work, the reliability of our simulations is assessed by using state-of-the-art universal MLIPs as well as systems with nominal charge neutrality, providing excellent agreement with benchmark ab initio simulations and experimental data (see Supplementary Section S1 in Supplementary Materials). With that, we prove that the traditional melt quench procedure is not applicable for modeling ALD alumina with high hydrogen contents, and propose an alternative equilibration procedure starting from highly defective bayerite structures annealed at experimental ALD temperatures (see Methods Section “Atomistic simulations”). The resulting structures closely match experimental stoichiometry and density measurements for ALD alumina polymorphs fabricated at different temperatures as shown in Fig. 2a. The corresponding XPS characterization in terms of Auger parameter shift has shown clear shifts in Al Auger parameters with ALD temperature, while no significant shifts were observed for the O Auger parameter (see Fig. 2b). Focusing further on Al Auger parameter shifts, the aim is to answer in this work if such shifts are attributed to the varying density, stoichiometry, or chemical states of individual elements. To do so, we combine experimental XPS characterization, high-fidelity atomistic simulations, ab initio calculations, and the electrostatic theory of extra-atomic relaxation, showing for the first time the possibility of probing chemical states of H with respect to its concentration in amorphous ALD alumina polymorphs.

## Results

### Structural analysis of amorphous ALD alumina

**Fig. 3:**
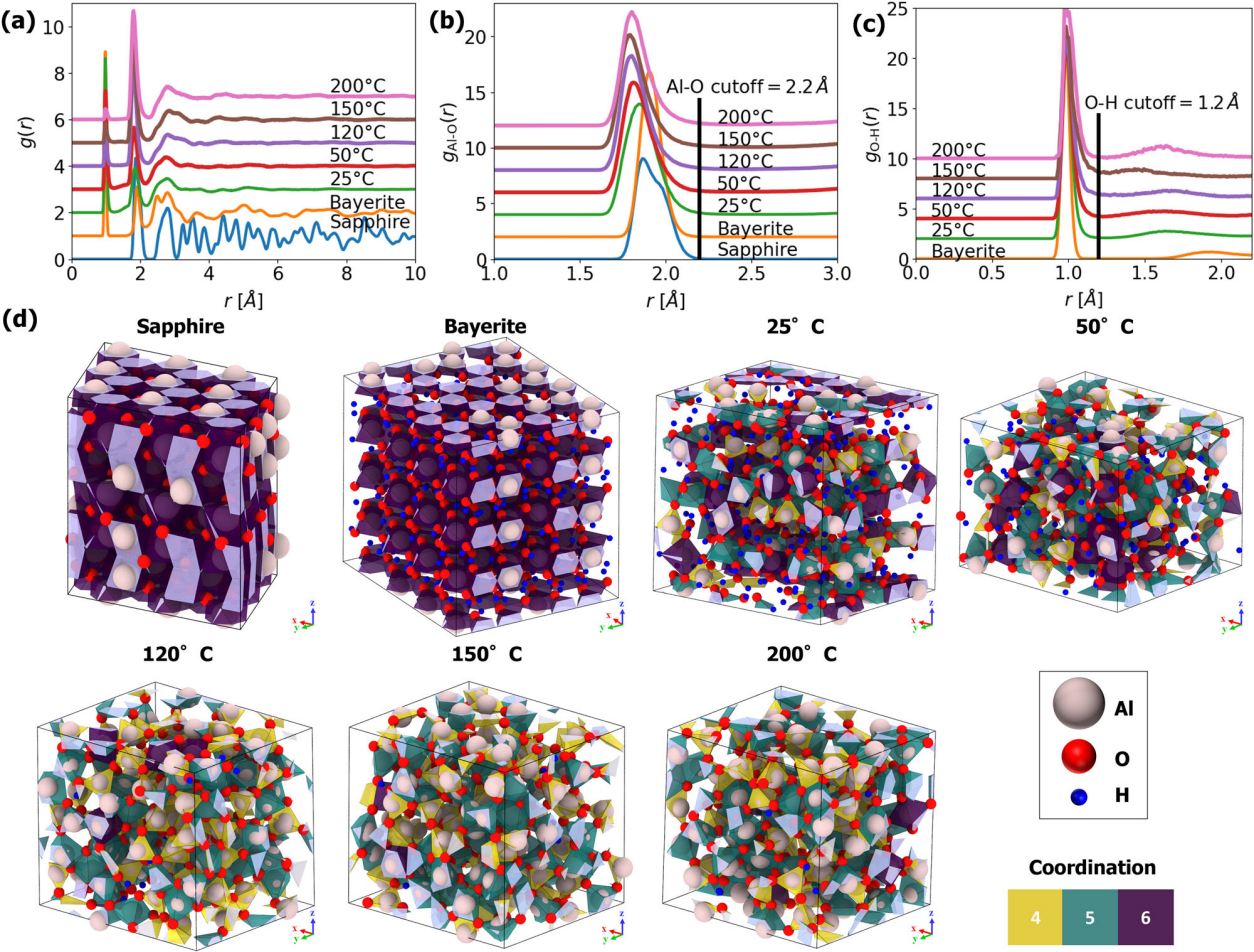
Coordination analysis of the amorphous ALD alumina samples. **a** The total radial distribution functions, *g*(*r*), showing long-range order in crystalline phases and its absence in amorphous polymorphs. **b** The partial Al-O radial distribution function, *g*_Al-O_(*r*), justifying the Al-O bond cutoff (black line). **c** The partial O-H radial distribution function, *g*_O-H_(*r*), justifying the chosen hydroxyl group cut-off (black line). **d** Analysis of five amorphous alumina polymorphs with different H/Al ratios and densities as well as of crystalline sapphire and bayerite. Details on simulation cell dimensions and atom counts appear in Supplementary Table S2.

Figure 3 represents a comprehensive structural analysis of five different amorphous oxide polymorphs with specific H/Al-ratios and densities, as aligned with the experiment (see Fig.2 a), shown together with two reference crystalline compounds. Details on the respective cell dimensions are given in Supplementary Table S1. The total radial distribution functions *g*(*r*) from *r* = 0−10 Å, as well as the bond-specific Al-O and O-H partial radial distribution functions *g*_*n*−*m*_(*r*) of atom *n*, *m*, from *r* = 0−3 Å are shown in Fig. 3a–c. The *g*(*r*), in Fig. 3a evidences long-range order for crystalline sapphire and bayerite, whereas the amorphous polymorphs only exhibit short-range order (i.e. a broad first nearest-neighbor peak only). Only bayerite shows a split of the O-O peak due to its two distinctly different oxygen environments^60^. The partial Al-O radial distribution function, *g*_Al-O_(*r*), in Fig. 3b (for *r* = 1−3 Å) displays a fairly narrow spread of the Al-O bond lengths for the different amorphous polymorphs; the first neighbor peak shifts from 1.93 Å to 1.85 Å when moving from the crystalline references to the amorphous polymorphs. Accordingly, a respective coordination cutoff radius of 2.2 Å (vertical black line, positioned well after the first nearest neighbor peaks) was selected for further structural analysis of the 6-fold, 5-fold, and 4-fold [AlO_4_] nearest-neighbor coordination spheres (NNCSs). The partial O-H radial distribution function *g*_O-H_(*r*) in Fig. 3c (for *r* = 0−2.1 Å) indicates a constant O-H bond length for all structures studied, in accordance with the presumed strong hydrogen bonding in forming hydroxyl groups. Accordingly, a respective coordination cutoff radius of 1.2 Å (vertical black line) can be used for determining the fraction of OH ligands in each amorphous polymorph. Figure 3d shows a first-neighbor shell coordination analysis of Al atoms. The crystalline *α*-Al_2_O_3_ reference (sapphire) is composed of Al^3+^ cations in 6-fold octahedral coordination with O^2−^. The crystalline *α*-Al(OH)_3_ trihydroxide reference (bayerite) is composed of a stacking of two planes of close-packed OH^−^ with Al^3+^ in octahedral coordination sandwiched between them. The amorphous structures generated at a process temperature of 25 °C display a more complex distribution of nearly equal fractions of 6-fold (violet), 5-fold (green) and 4-fold (yellow) [AlO_*n*_] polyhedra, in excellent agreement with experimental findings^61–66^. This implies that the artificially created defective bayerite structures (see Section “Atomistic simulations”) transform into an amorphous state during thermal equilibrium at the respective ALD growth temperatures. Comparison of the amorphous alumina polymorphs with different H/Al ratios and densities (corresponding to ALD temperatures of 50 °C, 120 °C, 150 °C, and 200 °C, respectively; as quenched to *T*_E_ = 300 K) reveals the following trend: an increase of the ALD processing temperature results in a lower hydrogen content, which is accompanied by a decrease of the proportion of 6-fold [AlO_*n*_] polyhedra, with 5-fold NNCS predominating over 4-fold ones.

**Fig. 4:**
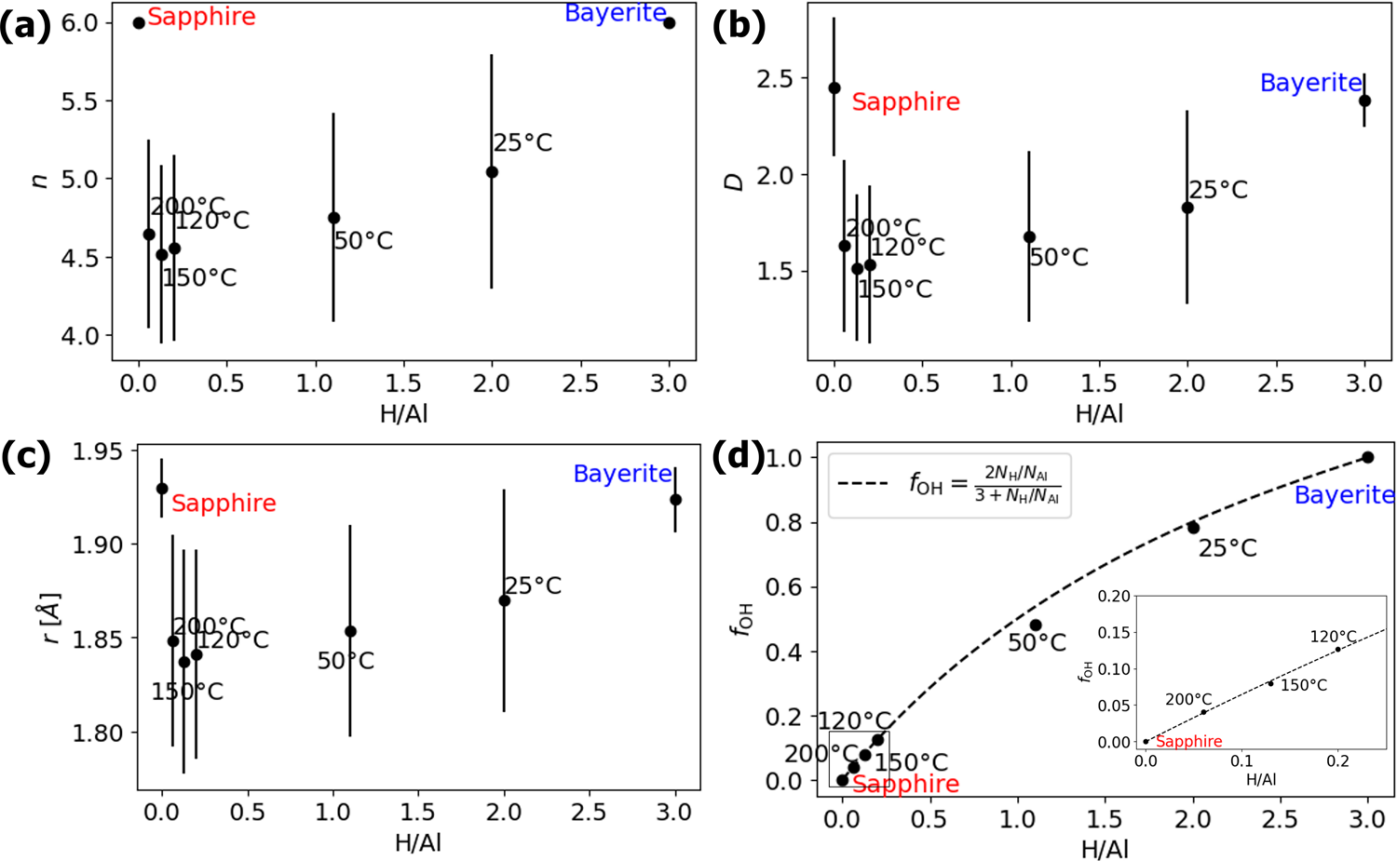
Estimating average structural features of ALD alumina layers. Average structural parameters (with corresponding standard deviation) as extracted from the Al NNCS of the simulated amorphous alumina polymorphs, as well as of the crystalline oxide and hydroxide reference phases shown as a function of H/Al ratio. **a** The average nearest-neighbor coordination number of Al cations by O(H) ligands, *n*. **b** The average geometric factor, *D*. **c** The average Al-O bond length, *r*. **d** The average fraction of OH ligands, *f*_OH_ (determined using a cutoff distance of 1.2 Å).

To provide more quantitative insights into the structure of amorphous oxides, we measure their structural descriptors as defined in the electrostatic model (Eq. (1)), averaged over structure and time. Figure 4 shows mean coordination *n*, geometric factor *D*, and bond length *r* (with their corresponding standard deviations) versus the H/Al-ratio, as extracted from the simulated structures of the amorphous alumina polymorphs as well as of the crystalline oxide and hydroxide reference phases. The average nearest-neighbor coordination number of Al cations by O(H) ligands, *n*, in Fig. 4a reveals a clear separation between the crystalline reference phases and the amorphous polymorphs. For sapphire and bayerite, all Al cations are in 6-fold coordination with O and OH ligands, respectively. The amorphous polymorphs result in lower average coordination numbers, which also exhibit a relatively large spread (i.e. a large standard deviation). This can be attributed to the characteristic network of randomly interconnected and partially distorted 4-fold, 5-fold and 6-fold [AlO_*n*_] polyhedral building blocks in amorphous alumina^61–67^.

The average *n*-value for the amorphous polymorphs decreases with decreasing H content (i.e., with increasing *T* except for the highest one), which is mainly due to a decreasing fraction of 6-fold NNCS (as compared to 4-fold and 5-fold NNCS; see Fig. 3a). This simulation result is in excellent agreement with the experiment showing that [AlO_6_] polyhedra convert to [AlO_5_] and [AlO_4_] polyhedra when releasing OH ligands and/or H_2_*O* during thermal annealing^64^. As discussed in ref. ^64^, hydroxide ligands in Al NNCS can convert to bridging O − H ⋯ O species, two of which can react to form a single Al-O-Al bridging configuration, thereby reducing the average coordination number of Al. Notably, this process is already thermally activated at temperatures as low as 200 °C^64^, which nicely coincides with the onset temperature for the crystallization of amorphous alumina in the range of 180–200 °C^50,68^. The early stage crystallization of amorphous alumina annealed at 200 °C may explain the corresponding anomaly breaking the general trend. While it would be beneficial to explore such phenomena further, the crystallization kinetics in oxides is extremely slow (seconds to hours)^50,68^, well beyond the limited timescales (nano- to microseconds) of molecular dynamics simulations. Figure 4b shows the corresponding average geometrical factors, *D*, as a function of the H/Al ratio. The crystalline phases exhibit the highest *D*-value close to 2.37, as calculated for perfect octahedral (6-fold) building blocks by Moretti^47^. The amorphous polymorphs all have a lower average *D* value due to the coexistence of 4-fold and 5-fold (besides 6-fold) NNCS. For all amorphous polymorphs, the average geometric factor exceeds *D* > 1.15, as calculated for a perfect tetrahedral (4-fold) NNCS^47^. In this regard, it is observed that some [AlO_*n*_] NNCS in amorphous alumina, and even in well-defined crystalline reference phases (such as *γ*-Al_2_O_3_ and even *α*-Al_2_O_3_) will be partially distorted^61,64,67^, which induces spread in *D* (and also of *r*; see below). As demonstrated in Supplementary Fig. S2, the established relation between *D* and *n* for the amorphous structures studied can be approximated by *D* = 0.07*n*^2^, which further simplifies Eq. (1) to:

3 $$\Delta \alpha \approx 2{R}^{{\rm{ea}}}\approx \frac{14.4n\zeta }{0.07{n}^{2}\zeta r+{r}^{4}}$$

Figure 4c shows the average radius, *r*, between the Al cations and their nearest-neighbor O(H) ligands (i.e. the average Al-O bond length) as a function of the H/Al ratio. The average Al-O bond lengths for sapphire and bayerite are very similar, which highlights the structural similarity of the long-range ordered [AlO_6_] and [Al(OH)_6_] NNCS in both phases. The amorphous polymorphs exhibit considerably lower average Al-O bond lengths (with a much larger variation), which complies well with the smaller average Al-O bond length for 4-fold (and presumably also of 5-fold) [AlO_*n*_] NNCS as compared to 6-fold NNCS^69^. The bond lengths in the range of 1.84–1.87 Å, as extracted from the simulated amorphous structures, are in excellent agreement with the experimental Al-O bond lengths for amorphous alumina ALD films in the range of 1.81–1.84 Å^70^. The amorphous polymorphs show a gradual decrease in the average length of the Al-O bond with decreasing H content (i.e. with increasing *T*_ALD_), in accordance with a simultaneous decrease in *n*, as discussed above. Consequently, the average Al-O bond distance, *r*, and the average coordination number, *n*, show a strong correlation, which can be expressed, for example, in the empirical power law as *r* = 1.4*n*^1/6^ (see Supplementary Fig. S2).

### Extracting ligand polarizabilities with electrostatic model

The observation that sapphire and bayerite have near identical values of *D*, *r*, and *n*, but display the 0.7 eV difference in their Al Auger parameters^42^ emphasizes strikingly different electronic polarizability for the O and OH ligands, respectively. Such a difference can be justified by calculating the charge isosurfaces of O in sapphire and bayerite (by ab initio calculations; see Methods), which is shown in Supplementary Fig. S3a and b, respectively. It follows that for sapphire, the valence charge of O is near spherical and slightly stretching towards three Al atoms, forming a trigonally elongated spherical shape typical for each O ligand. In contrast, for bayerite, there is a notable shift in the O valence charge distribution towards the H atom (due to the formation of covalent O-H hydroxyl bonds), resulting in a more drop-like shape of the charge density for OH ligands.

To simplify the further analysis, we assume that the average ligand polarizability follows the rule of mixture:

4 $$\zeta ={\zeta }_{{\rm{O}}}(1-{f}_{{\rm{OH}}})+{\zeta }_{{\rm{OH}}}{f}_{{\rm{OH}}}$$

where *ζ*_O_ and *ζ*_OH_ are unknown individual ligand polarizabilities, and the fraction of OH ligands *f*_OH_ can be derived from the simulated structures using a cutoff of 1.2 Å as derived in Fig. 4d. The resulting fractions of OH ligands are plotted as a function of the H/Al ratio in Fig. 4d. The strict constraint of overall charge neutrality demands the following relation between the fractions of cations and anions: 3*N*_Al_ − 2*N*_O_ − *N*_OH_ = 0. If all hydrogen is assumed to react with O to form OH ligands (i.e. *N*_OH_ = *N*_H_)^42,71,72^, the following relation between the fraction of OH ligands and the H/Al ratio is obtained:

5 $${f}_{{\rm{OH}}}=\frac{{N}_{{\rm{OH}}}}{{N}_{{\rm{O}}}+{N}_{{\rm{OH}}}}=\frac{2{N}_{{\rm{H}}}/{N}_{{\rm{Al}}}}{3+{N}_{{\rm{H}}}/{N}_{{\rm{Al}}}}$$

The dashed line in Fig. 4d indicates this relationship and shows very good agreement with the simulated data points, which supports our assumption that H preferentially bonds to O ligands of the Al cations, thereby reducing the average number of O ligands with respect to OH ligands. It is important to note that minor discrepancies from the optimal relationship can be attributed to the dynamic nature of O-H bonds, which are influenced by thermal fluctuations. This phenomenon may result in an overestimation of the fraction of hydroxyl groups due to the co-existence of “non-bonded" (i.e., more mobile) interstitial protons. This topic will be discussed in further detail later on.

**Fig. 5:**
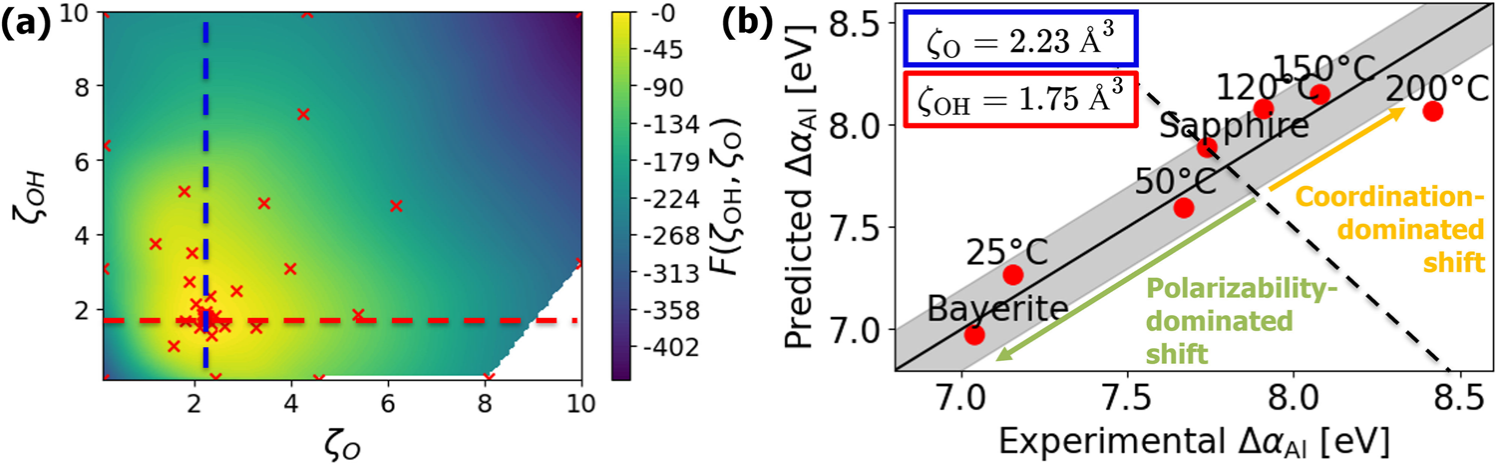
Derivation of ligand polarizabilities from Auger parameter shifts. **a** Visualization of the Bayesian optimization procedure over 104 iterations to determine optimal ligand polarizabilities. **b** Validation plot comparing predicted vs. experimental Al Auger parameter shifts, using the Al atomic gas reference *α*_Al,gas_ = 1454.0 eV from Moretti^103^. The gray region indicates the empirical experimental uncertainty of ±0.2 eV.

The unknown polarizabilities of O and OH ligands were derived next by superimposing experimental data and Eqs. (1) and (5), and using a Bayesian optimization process (see Methods) employing the extracted average structural parameters pertaining to Fig. 4a–d. Figure 5a demonstrates that the mixed exploration/exploitation procedure in the Bayesian optimization of *ζ*_O_ and *ζ*_OH_ rapidly converges to values within the range of 0.9–3.2 Å^3^, in agreement with experimental range for O ligands in various compounds^73,74^. The optimization process results in optimized values of *ζ*_OH_ = 1.753 Å^3^ and *ζ*_O_ = 2.227 Å^3^, which match well a rough empirical estimate of 2 Å^3^ for free-standing OH^−^ and O^2−^ ligand polarizabilities in ref. ^75^.

In a final step, the Al Auger parameter shift was calculated from Eq. (1) using the optimized *ζ*_O_ and *ζ*_OH_ ligand polarizabilities. The predicted values are plotted against experimentally measured Auger parameter shifts in Fig. 5b, evidencing an excellent agreement between experiment and theoretical prediction, highlighting the applicability of Eq. (1) to amorphous materials when the structure-averaged descriptors of local atomic environments are used. Furthermore, by using sapphire as a reference, we can clearly identify the dominating factors for measured chemical shifts. While coordination reduction associated with amorphization increases the corresponding Auger parameter, the hydrogen incorporation lowering the average ligand polarizability reduces the Auger parameter. Thus, amorphous alumina with undercoordinated Al and no hydrogen incorporation sets the upper bound on Al Auger parameter shift, while bayerite with 6-coordinated fully hydroxylated Al sets the lower bound on Al Auger parameter shifts, with all other Al-O-based compounds falling in between.

### Chemical state analysis with ab initio calculations

**Fig. 6:**
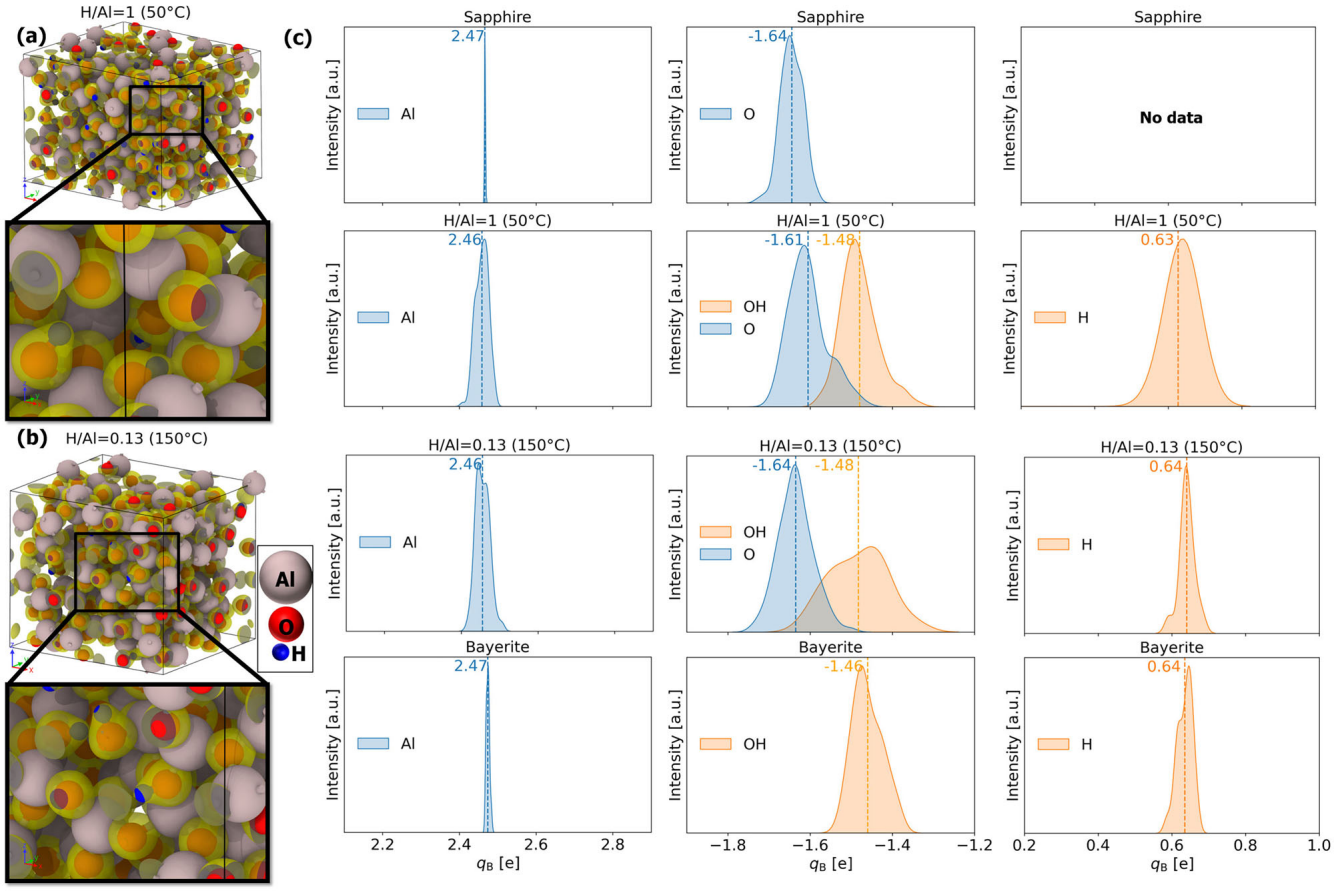
Ab initio chemical state analysis of H-supersaturated samples. Reconstructed isosurfaces of oxygen valence electrons for (**a**) an H/Al = 1 and (**b**) an H/Al = 0.13 amorphous polymorphs, showcasing the spatial distribution and density. **c** Kernel density estimates of Bader charges, *q*_B_, for aluminum, oxygen, and hydrogen atoms, organized into three respective columns. The mean values of the *q*_B_ distributions are indicated by the dashed lines.

Despite the demonstrated success, it is important to note that electrostatic models typically assume that extra-atomic relaxation is only influenced by the nearest-neighbor ligands with well-defined polarizabilities, independent of ligand environment. To validate this assumption, additional DFT calculations were performed to determine the charge densities of the valence electrons in Al, O, and H atoms, and their corresponding *q*_B_. Figure 6a, b shows visualizations of the isosurfaces around O in samples with H/Al ratios of 1 and 0.13, respectively (for which the calculated *Δα* closely matches the experimental values; see Figure 5b). Comparisons of these isosurface shapes with those of the crystalline reference phases reveal similar shapes and characteristics: compare Fig. 6a, b with Supplementary Fig. S3a, b. This supports our assumption that localized electronic screening of core-ionized Al atoms by nearest-neighbor O and OH ligands in the studied amorphous alumina polymorphs is very similar to those in the crystalline oxide and hydroxide reference phases.

Further validation is provided in Fig. 6c, which shows the distributions of the *q*_B_, for Al and H atoms, as well as for O atoms with and without H neighboring atoms in sapphire, bayerite, and in H-rich and H-poor amorphous polymorphs (i.e. for H/Al = 1 and H/Al = 0.13, respectively). Al cations exhibit relatively narrow distributions of *q*_B_ compared to O, being extremely narrow for the crystalline references. The amorphous polymorphs display slightly broader *q*_B_ distributions for Al. This indicates that the valence charge density of Al is only very slightly affected by hydrogen bonding to nearest-neighbor O ligands. Accordingly, the mean *q*_B_ values for Al are similar for all studied structures, ranging approximately from 2.46e to 2.47e in agreement with ref. ^76^. Particularly interesting are the *q*_B_ distributions for O ligands with and without H neighbors: see central column of Fig. 6c. It shows that O atoms without neighboring H have a mean *q*_B_ similar to that of sapphire, while O atoms with neighboring H have a mean *q*_B_ similar to that of bayerite. This nicely demonstrates why the adopted model and assumptions are so successful in predicting *Δα*_Al_ as a simple function of the fraction of hydroxyl ligands in amorphous polymorphs. The corresponding mean values of *q*_B_ for H atoms are also similar for both amorphous polymorphs and bayerite.

As reflected in Fig. 4a–c, the amorphous polymorphs exhibit similar trends in their structural parameters as a function of H content, except for the amorphous polymorph with the lowest H content, corresponding to the highest ALD temperature of 200 °C. In this regard, it is emphasized that this amorphous alumina ALD film is superimposed to thermal annealing at 200 °C during its 4 h deposition run^42^. As mentioned above, this highest ALD growth (and annealing) temperature exceeds the onset temperature for the crystallization of amorphous alumina in the range of 180–200 °C^50,68^. This suggests that the simultaneous thermal annealing of the developing ALD film at 200 °C is accompanied by slow but gradual crystallization, as evidenced by previous studies^50,68^. Such a phenomenon is likely to be more pronounced in the initially deposited, interface-adjacent regions of the amorphous alumina film. This crystallization will be accompanied by a gradual release of hydroxyl ligands and a modification of the isolated [Al(O)_*n*−1_(OH)] building blocks in the amorphous alumina film^64^. Indeed, the *n*, *D*, and *r* values all sharply increase towards those for sapphire for an increase of the ALD deposition temperature from 150 °C to 200 °C. Consequently, our presumption that all H impurity atoms are bonded to O in the form of hydroxyl ligands may be violated for the ALD film grown at 200 °C. This rationalizes the underestimate of *Δα*_Al_ with respect to *Δα*_Al_ for *T* = 200 °C in Fig. 5b.

**Fig. 7:**
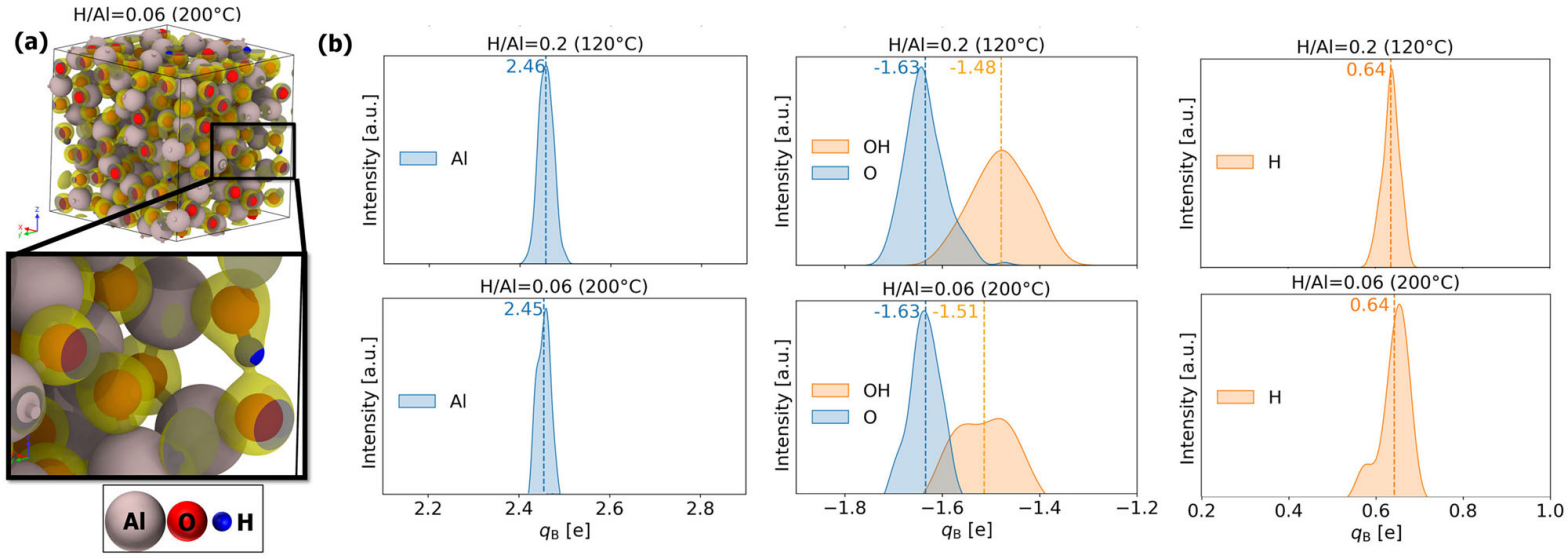
Ab initio chemical state analysis of low-H samples. Panels **a** depict the isosurfaces of oxygen valence electrons for H/Al = 0.06, showcasing the spatial distribution and density. Panel **b** presents kernel density estimates of Bader charges, *q*_B_, for Al, O, H atoms, organized into three respective columns. The mean values of the distributions are given and indicated as dashed lines.

This hypothesis is investigated in more detail in Fig. 7, which shows the distributions of the *q*_B_, for Al and H atoms, as well as for O atoms with and without H neighboring atoms, below and above the onset of the crystallization temperature range (i.e. for H/Al = 0.2 and H/Al = 0.06 pertaining to an ALD deposition temperature of 120 °C and 200 °C, respectively). Two distinct peaks for the OH ligands are visible for *T* = 200 °C, one having a mean *q*_B_ similar to bayerite and the amorphous polymorph for *T* = 120 °C (H/Al = 0.2). The other peak has a significantly lower mean, shifting the overall mean of *q*_B_ towards −1.51. This broadening by splitting-up of the *q*_B_ distribution for O may be tentatively contributed to the reduction of free volume and the development of long-range ordering between the interconnected [AlO_*n*_] polyhedral building blocks upon crystallization^50,68^, as accompanied by a gradual transformation of isolated hydroxyl species into O ligands and interstitial protons and/or O − H ⋯ O bridging configurations^71,72,77^.Notably, a similar broadening by splitting-up of the *q*_B_ distribution appears to evolve in the amorphous polymorph for *T* = 150 °C (H/Al = 0.13; see Fig. 6b, albeit less pronounced as for *T* = 200 °C. Consequently, the mixing rule used to fit *ζ* values for calculating *Δα* becomes invalid. The considerable underestimation of *Δα*_Al,s_ with respect to *Δα*_Al_ for *T* = 200 °C in Fig. 5b would imply an underestimation of the ligand polarizability of core-ionized Al ions for the amorphous polymorph at 200 °C.

In summary, we successfully resolved the structure and chemistry of amorphous ALD alumina polymorphs using (1) a novel approach to amorphous structure generation guided by experimental observations and (2) near-ab initio-accurate neural network interatomic potential. We demonstrate that the nature of H impurities in such amorphous compounds can be revealed by comparing the experimental Auger parameter shifts with those predicted from simple electrostatic theory by assuming that a dominant fraction of incorporated hydrogen atoms participates in OH hydroxyl bonds. Any deviation between experiment and theory beyond the experimental error indicates the co-existence of different H bonding states, such as interstitial H protons and/or O − H ⋯ O bridging configurations that appear at low H contents in ALD alumina films. Furthermore, the carefully resolved structure of amorphous oxides enables ab initio analysis in terms of equilibrium charge density profiles and partial Bader charges, providing further insights into the chemistry of these complex H-containing compounds. Such fundamental knowledge could contribute to advancing the design of hydrogen barrier films, hydrogen separation membranes, hydrogen storage materials, and fuel cells, through ALD routes, which deserves further exploration and discussion.

## Discussion

ALD is emerging as an effective method for coating custom-shaped metallic substrates. The primary requirement for effective protective coatings is robust adhesion to the substrate and maintaining integrity under variable temperatures. This necessitates the coating’s ability to handle thermal stress, which arises from the significant difference in thermal expansion coefficients between metallic substrates and ceramic coatings^78,79^. In this context, amorphous alumina offers an excellent balance of strength and ductility across all deformation modes i.e., tension, compression, and shear^80^. Atomistic simulations by Lee et al. have demonstrated that the exceptional strength and ductility of amorphous alumina, surpassing those of other amorphous oxides like titania and silica, are attributed to the density and dissociation energy of the metal-oxygen bonds^81^. Specifically, a higher volumetric density of these bonds contributes to increased yield and flow strengths, while a lower bond dissociation energy enhances bond-switching capability, thereby improving ductility.

Our findings suggest that increasing hydrogen content significantly weakens ALD alumina polymorphs. The formation of hydroxyl groups disrupts the …-Al-O-Al-… network connectivity, replacing dense -O-Al-O- bonds with weaker -O-H ⋯ O- bonds. This interaction also increases the free volume, consequently reducing the density of ALD alumina polymorphs at higher hydrogen levels. Since hydrogen content inversely correlates with the ALD process temperature^41^, raising the temperature should enhance the strength and Young’s modulus of ALD alumina films, aligning with experimental observations^82^. However, to confirm these hypotheses, it is crucial to explore further the stress relaxation mechanisms facilitated by hydrogen, as the volumetric bond density criterion proposed by Lee et al. does not consider local bond heterogeneities introduced by hydrogen^81^. Similarly, the prediction of ductility remains uncertain, as it is unclear how hydrogen and hydroxyl groups impact the bond-switching mechanism in amorphous ALD alumina films. Experimental studies have shown a reduction in crack onset strain with lower ALD temperatures (i.e., higher hydrogen content)^43,81^ but, due to large overlapping error bars in these studies, it is challenging to definitively attribute this trend to hydrogen effects rather than a general decrease in the density of amorphous alumina due to reduced ALD temperatures. As discussed in refs. ^43,78^, the residual thermal stress after cooling the film from the ALD process temperature to room temperature could also influence the crack onset strain. Additionally, the high atomic mobility of hydrogen at room temperature likely promotes hydrogen segregation to stress concentration sites, potentially facilitating nanopore formation and crack nucleation. These aspects warrant further investigation through atomistic simulations to clarify hydrogen’s role in the mechanical performance of ALD alumina films.

On the other hand, understanding hydrogen incorporation and diffusion in alumina coatings is critical for designing effective hydrogen permeation barriers, particularly for metals used in hydrogen gas storage and nuclear fusion reactors^1^, where advanced metallic materials are prone to hydrogen embrittlement-a phenomenon distinct from classical corrosion in oxygen and water environments^83,84^. Although alumina is an efficient hydrogen permeation barrier, its efficacy is mostly demonstrated in crystalline thin film coatings^10^. The atomistic modeling community commonly uses amorphous alumina to represent crystalline alumina grain boundaries at elevated temperatures^72,77^. The closest matches to experimental activation energies in such studies are found for proton (H^+^) diffusion bonded to the nearest oxygen atoms, with diffusion primarily involving bond switching, aligning with the observations and expectations obtained from prior DFT calculations^71^. However, these studies have only investigated very low hydrogen contents in small supercells due to the limitations of DFT and ab initio MD. A Gaussian approximation MLIP was applied solely to extend simulation timescales^72^. Thus, systematic studies on the effect of hydrogen content on its diffusion coefficients and activation energies remain a pressing matter for future research. The presented results indicate a significant change in the chemical state of hydrogen at around 4.7 at.% H (H/Al ratio of 0.13), likely altering the diffusion mechanisms due to the increasing role of hydrogen-hydrogen interactions.

Although the solubility of hydrogen in amorphous alumina has never been systematically studied, it should be relatively low based on experimental^85^ and theoretical^86^ results for crystalline alumina, with the highest concentrations reaching about 0.17 at.% at surfaces, grain boundaries, and cracks^87^. Substantially higher H contents (although surveyed as D) of 2–4 at.% have been observed in the aluminum oxide layer formed during corrosion of an Al alloy, primarily attributed to Mg segregation in the oxide layer^88^. Thus, it can be confidently stated that the ALD amorphous alumina polymorphs in this study, with H compositions ranging from 2.4 to 36.4 at.%, represent supersaturated metastable phases that are kinetically stabilized mostly due to hydrogen trapping within the films at low temperatures. It is unclear whether such supersaturated states would serve as beneficial hydrogen permeation barriers in different environments and with various metallic substrates, highlighting the need for further research. For instance, such supersaturation may promote hydrogen segregation to the coating-substrate interface, reducing their adhesion^89^. However, it may also inhibit further hydrogen adsorption, splitting, and incorporation when exposed to hydrogen gas at high pressures, thus providing a higher permeation ability that could be comparable to or even exceed that of so-far-tested crystalline alumina coatings.

However, for thin coatings combined with strong tensile strains associated with the ALD process^90^, such supersaturation might lead to a nanoporous structure beneficial in other areas of the hydrogen economy, such as designing nanoporous oxide membranes for hydrogen gas separation^91,92^. Experimental characterization of such nanoporosity is challenging, so follow-up computational studies of biaxial residual stress-driven nanoporosity formation in ALD alumina polymorphs at different temperatures and hydrogen contents would shed light on numerous experimental observations, also related to the application of ALD alumina overcoats in catalysis^92^. As an example, we heated up the H-rich H/Al = 2 sample from 300 to 1000 K for one nanosecond at zero pressure along one dimension to mimic the presence of a free surface in the thin film and then annealed the sample at 1000 K for another one nanosecond. We added Supplementary Video (SupplVideo.mp4) demonstrating the formation of nanoporous amorphous alumina film during sample annealing, with the resulting structure made of intercalated nanopores filled with water and alumina scaffold covered with hydroxyl groups. The latter is expected to prevent pores from collapsing after water finds its way towards the free surface and leaves the scaffold. Further research will reveal the specifics of such phenomena, potentially enabling the design of next-generation gas separation membranes through ALD processing routes.

## Methods

### Atomistic simulations

In our study, the methodology used was centered on molecular dynamics (MD) simulations, a technique that is pivotal to probing the atomic-scale interactions and structural nuances of materials. The primary focus was to elucidate the effects of hydrogen incorporation within amorphous alumina films on the corresponding experimentally measured Auger parameter shifts. MD simulations were carried out using Large-scale Atomic/Molecular Massively Parallel Simulator (LAMMPS)^93^. The Nosé–Hoover thermostat and the Parinello–Rahman barostats were used for temperature and pressure control with damping coefficients of 0.05 ps and 0.5 ps, respectively. A timestep was set to 0.5 fs, smaller than the standard value to avoid artifacts related to unrealistic hydrogen mobility in amorphous structures, and to ensure proper equipartition between different degrees of freedom in the system^94^. Periodic boundary conditions were applied along all dimensions to model bulk phases, excluding surface effects.

After the simulation phase, structural adjustments and analyses were performed using pymatgen^95^, Atomic Simulation Environment (ASE)^96^, and the OViTo visualization tool^97^. ASE and pymatgen offer a robust framework for manipulating atomic simulations, enabling straightforward structure modifications, property calculations, and result visualizations. In contrast, OViTo^97^ provides sophisticated visualization and analysis capabilities, especially through its Python interface, making it possible to render and analyze complex atomic structures in an intuitive and detailed manner.

For MD simulations, a sophisticated universal graph neural network potential, specifically PreFerred Potential (PFP) *v5.0.0 CRYSTAL_U0_PLUS_D3*^59^, was used. We recently demonstrated the applicability of this type of graph neural network potentials for the accurate modeling of amorphous alumina^56^. PFP stands out because of its ability to simulate a broad spectrum of molecular and crystalline systems, extending its utility to uncharted materials. It supports simulations that involve combinations of up to 72 elements, supported by a comprehensive training dataset comprising more than 32 million structures consistently derived from high-quality density functional theory (DFT) calculations^98^. As with any other potential, its applicability to the system and property of interest is first tested and justified, as discussed in Supplementary Section S1. After the inclusion of Van der Waals dispersion correction, the potential accurately reproduces the structures and properties of reference crystalline phases and liquid alumina. While the PFP model does not impose formal restrictions on system composition or stoichiometry due to the exclusion of atomic charges from energy and force estimation, we have enforced nominal charge neutrality in these and further calculations to enhance their reliability. This approach may result in minor deviations from experimentally derived stoichiometries for ALD aluminas, but it is a necessary compromise to ensure the accuracy of our results.

**Fig. 8:**
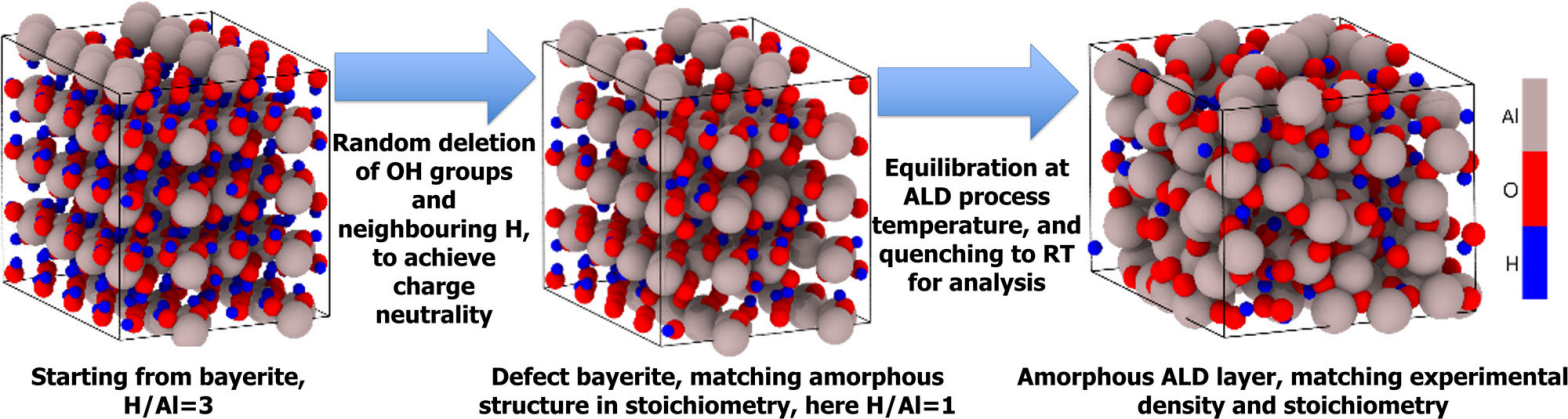
Summary of amorphous alumina generation procedure. A schematic representation of the novel simulation method employed to generate amorphous oxide polymorphs with different H-contents and densities by starting from an artificially constructed, highly defective bayerite structure, while maintaining nominal charge neutrality.

In the first attempt, amorphous alumina polymorphs with different H contents were simulated using the traditional melt quenching procedure. Specifically, we started with a sapphire crystal containing 270 atoms, heated it beyond its melting point to 3500 K, annealed it until the structural factor stopped changing, and quenched it to room temperature with ≈20 K/ps with all the steps done at zero pressure. To reach the targeted H content from the experiment, we tried to randomly introduce H atoms into liquid or amorphous alumina, ensuring that they are positioned at a reasonable distance from the surrounding atoms. To maintain charge neutrality within the modeled systems, Al atoms were randomly removed in proportion to the number of inserted H atoms, adhering to a ratio of three H atoms for every Al atom removed. However, for H/Al ratios >1, the random insertion of hydrogen resulted in proximal H atoms forming H_2_ molecules that normally should not be present at any stages of the ALD process^41,42^. Unfortunately, this issue could only be partially mitigated by selecting various random starting configurations. Moreover, if H is inserted into the liquid alumina before quenching, water molecules can emerge even at a low H content. Although water is an essential part of the ALD process, subsequent XPS analysis of ALD alumina samples did not show any signs of residual water embedded in the structure^42^. *We thus concluded that the traditional melt quenching procedure is not suited to generate representative amorphous alumina polymorphs with different H-contents, as observed in the experiment*. Therefore, *a novel approach*, visualized in Fig. 8, was attempted to simulate amorphous oxide polymorphs with different H contents while ensuring a good match with the experimental densities.

Based on experimental knowledge that H atoms in amorphous oxides strongly interact with oxygen anions to form hydroxyl ligands^42,72^, H atoms were not randomly inserted into the simulation samples but instead embedded as hydroxyl groups from the start of the simulations. To this end, the Al-trihydroxide phase, bayerite, was adopted as the starting crystalline structure. The OH groups and neighboring H atoms were then randomly removed, mimicking the dehydration of bayerite. The removal of equal amounts of OH and H pairs ensured charge neutrality, while the resulting H/Al ratio was matched to the experiment by adjusting the number of removed pairs. Next, the dimensions of such defective bayerite structure were scaled down to achieve a density set approximately 10% higher than the corresponding experimental value to promote interactions between neighboring Al and O atoms with broken bonds. The resulting structure was equilibrated in NVT (constant number of atoms N, constant volume V, constant temperature T) ensemble for 50 ps at the corresponding *T*_ALD_. This equilibration phase induced the transformation of the structurally unstable, highly defective bayerite into an amorphous state. Subsequently, the amorphous structure was quenched from *T*_ALD_ to the equilibration temperature, *T*_E_ = 300 K, for an additional 50 ps in the N*p*T (with constant pressure *p*) ensemble, during which all six dimensions of the cell (i.e., size and shape) were allowed to relax independently to zero stress. Following the quench, a further 50-ps equilibration at *T*_E_ = 300 K in the same ensemble was performed, during which the cell dimensions were sampled every 0.005 ps and then averaged. The final production run was performed at *T*_E_ = 300 K in the NVT ensemble, with the cell dimensions and tilt angles fixed to these averaged values.

Scripts and trajectories are publicly available at materialscloud.org^99^. The resulting densities are reported in Supplementary Table S1, demonstrating excellent agreement between the densities predicted from simulations and measured experimentally (see Fig. 2), highlighting the reliability of PFP. A comparable impressive agreement is achieved for the crystalline reference structures using the two equilibration stages performed at *T*_E_ = 300 K. In the final production run, 1000 snapshots (i.e. every 100 timesteps) of each system are generated for the time-averaged analysis of sample structures accounting for thermal noise.

### Bayesian optimization of ligand polarizabilties

Bayesian optimization is a powerful technique for optimizing objective functions that are expensive to evaluate. It operates by constructing a posterior distribution of functions, typically using a Gaussian process, which serves as a probabilistic model for predicting the objective function’s outputs based on its inputs. As observations accumulate, this posterior distribution is refined, enhancing the algorithm’s capacity to discern promising regions within the parameter space for further exploration. For a comprehensive understanding of this method, readers are referred to the relevant literature^100–102^.

By applying the formalism of Eqs. (1) and (4) to 1000 representative snapshots from each sample’s MD production run, we obtain time- and structure-averaged Al Auger parameter shifts (*Δα*_Al,pred_). Using the reference Al atomic gas value *α*_Al,gas_ = 1454.0 eV from Moretti^103^, these predicted shifts are then compared to the experimentally measured values, *Δα*_Al,exp_, as shown in Figure 2. This formulation enables the establishment of an acquisition function, *F*, which solely depends on *ζ*_OH_ and *ζ*_O_, expressed as:

6 $$F=-\sum _{\,\text{samples}\,}{\left(\Delta {\alpha }_{{\rm{Al,exp}}}-\Delta {\alpha }_{{\rm{Al,pred}}}({\zeta }_{{\rm{OH}}},{\zeta }_{{\rm{O}}})\right)}^{2}.$$

This function *F* is then maximized using Bayesian optimization (with the Python code^104^) within the experimentally reported limits for the polarizability volumes of metal oxides between 0.9 and 3.2 Å^3^ set equal for both ligands^73^.

### Ab initio calculations

To interpret the extracted polarizabilities, DFT calculations with ground-state Bader charges, *q*_B_, analyses were performed in reference crystalline compounds and amorphous samples using Vienna Ab initio Simulation Package (VASP) software (version 6.3.0)^105–108^. Core electronic interactions were modeled using the projector augmented-wave (PAW) method to treat the electron-ion interactions within a plane-wave basis^109,110^. The exchange-correlation energy was approximated by the generalized gradient approximation (GGA)^111^, employing the PBE functional to balance computational efficiency with precision in material property predictions^112^. Brillouin zone sampling was restricted to the *Γ*-point only, which is a reasonable assumption due to the relatively large simulation cells (over 300 atoms) used in this work.

The treatment of Van der Waals interactions, crucial for precise intermolecular force calculations, was incorporated through the DFT+D3 method^113^. *q*_B_ analysis was conducted to determine atomic charges using algorithms and methods developed by Henkelman et al.^114–117^. The calculation of *q*_B_ plays a crucial role in understanding and predicting the chemical shifts observed in spectroscopic methods like XPS and Auger spectroscopy. By establishing a linear relationship between the Auger parameter shift and the ground state Bader valence charge, researchers can now separate the initial and final state effects that co-determine core electron binding energy shifts^118^. This method provides a robust framework for rationalizing the variance in binding and Auger energies across different compounds, particularly halides, and chalcogenides of Ba, Zn, Pb, and Cu^119^. Future research can leverage this approach to quantify and isolate factors affecting the kinetic energies of core photoelectrons, enhancing the accuracy of spectroscopic analysis and potentially revealing unique electronic properties of materials, including semiconductor behaviors.

The initial structures for calculating *q*_B_ were derived from the last snapshot of the MD production simulations with PFP, which then undergoes the relaxation of the ionic positions using VASP using a conjugate gradient minimization while maintaining the shape and size of the simulation box. This allows us to remove thermal fluctuations before analysis while retaining the *T*_E_ = 300 K density of each system. Due to the large size of the simulation box, the 25 °C ALD sample analysis was not used with DFT. For the same reason, a smaller cell for bayerite with 112 atoms instead of 896 atoms is introduced and used for the *q*_B_ analysis.

## Supplementary information

Supplementary Information

Supplementary Video

## Data Availability

Data supporting the findings of this study are openly available at the following URL/DOI: 10.24435/materialscloud:9v-61.
