# Supplementary material for: Unveiling hydrogen chemical states in supersaturated amorphous alumina via machine learning-driven atomistic modeling
Source: NPJ Comput Mater. 2025 Jun 6;11(1):170. doi: 10.1038/s41524-025-01676-5 (PMC12143980; doi:10.1038/s41524-025-01676-5)
Supplement: Supplementary file 1 — Supplementary Information [file 41524_2025_1676_MOESM1_ESM.pdf]

Supplementary Materials: Unveiling hydrogen chemical states in supersaturated amorphous alumina via machine learning–driven atomistic modeling

Simon Gramatte[1,2,3,4], Olivier Politano[2], Noel Jakse[5], Claudia Cancellieri[3], Ivo Utke[6], Lars P.H. Jeurgens[3], and Vladyslav Turlo[1,4,*]

[1]Laboratory for Advanced Materials Processing, Empa - Swiss Federal Laboratories for Materials Science and Technology, Feuerwerkerstrasse 39, 3602 Thun, Switzerland

[2]Laboratoire Interdisciplinaire Carnot de Bourgogne, UMR 6303 CNRS-Université Bourgogne Europe, 9 Avenue A. Savary, 21078 Dijon Cedex, France

[3]Laboratory for Joining Technologies and Corrosion, Empa - Swiss Federal Laboratories for Materials Science and Technology, Ueberlandstrasse 129, 8600 Duebendorf, Switzerland

[4]National Centre for Computational Design and Discovery of Novel Materials MARVEL, Empa, Thun, Switzerland

[5]SIMaP, Grenoble-INP-UGA, CNRS, University of Grenoble Alpes, 38042 Grenoble, France

[6]Laboratory for Mechanics of Materials and Nanostructures, Empa - Swiss Federal Laboratories for Materials Science and Technology, Feuerwerkerstrasse 39, 3602 Thun, Switzerland

[*]Corresponding author: Vladyslav Turlo, vladyslav.turlo@empa.ch

April 16, 2025

S1 Interatomic Potential Justification

We begin our tests with the comparison of the equilibrium structures of sapphire and bayerite. The initial configurations have been taken from the Materials Project (`next-gen.materialsproject.org/materials/mp-1143`, `next-gen.materialsproject.org/materials/mp-560572`). These configurations underwent energy minimization with PFP in LAMMPS (energy tolerance 10^{-4} eV) and force tolerance (10^{-6} eV/Å) with box relaxation to zero values of the six components of the pressure tensor. In Table S1, we directly compare the unit cell vectors and densities of our simulated structures with the values obtained from DFT calculations and experimental data. PFP is designed to predict the energies and forces derived from DFT computations with Projector augmented wave (PAW) pseudopotentials Perdew-Burke-Ernzerhof (PBE) functional. Thus, it demonstrates quite a good accuracy when compared to DFT results, but it does not perform well against experimental data.

Despite the broad applicability of DFT across various material systems, it encounters limitations in accurately capturing certain phenomena, notably those involving dispersion forces (Van der Waals forces). These forces play a significant role in simulations in which dipole interactions, stemming from electronic state excitations, are critical to capture the properties of oxides, hydroxides, and especially layered materials [1]–[4]. The primary focus of DFT on the electronic ground state presents challenges in directly addressing dispersion forces. To mitigate these limitations, the DFT-D3 method emerges as a prevalent solution, effectively accounting for the dispersion force

contributions while combined with the PBE pseudopotential [5], [6]. This approach involves the explicit computation of the dispersion force energy (D3 correction) and its subsequent addition to the DFT-derived energy [7]. This synergy allows for a more nuanced simulation of material behavior, bridging the gap between theoretical accuracy and computational efficiency.

Leveraging the torch-dftd framework [8], the PFP model incorporates the D3 correction to improve its accuracy in simulating material properties with a marginal increase in computational time. As shown in Table S1, PFP+D3 matches better experimental densities, cell vectors a, b, c, and cell angles α, β and γ as compared to PFP. This evidence strongly advocates for the utilization of PFP+D3 in current modeling efforts, especially in contexts where accurate reproduction of experimental results is critical.

Table S1: Comparison of different properties between crystalline reference structures of bayerite and sapphire, juxtaposed with experimental and ab initio data. The computational models include structures obtained through PFP+D3, PFP, and DFT modeling. These computational results correspond to structures stabilized at $T = 0$ K. In contrast, the experimental data were acquired at 25°C, offering a relevant comparison across different conditions and methodologies.

Property	Bayerite				Sapphire			
	PFP+D3	PFP	DFT	Exp. (25°C)	PFP+D3	PFP	DFT	Exp.
ρ [g/cm^3]	2.50	2.36	2.39 [9]	2.50[10], [11]	3.90	3.82	3.87 [12]	3.9-3.98 [10], [13]
a [Å]	5.08	5.15	5.15 [9]	5.06 [11]	4.80	4.82	4.81 [12]	4.76 [13]
b [Å]	8.73	8.84	8.92 [9]	8.68 [11]	4.80	4.82	4.81 [12]	4.76 [13]
c [Å]	9.34	9.63	9.42 [9]	9.42 [11]	13.08	13.17	13.12 [12]	13.00 [13]
α [°]	90.00	90.00	90.00 [9]	90.00 [11]	90.00	90.00	90.00 [12]	90.02 [13]
β [°]	90.41	90.26	90.49 [9]	90.49 [11]	90.00	90.00	90.00 [12]	89.98 [13]
γ [°]	90.00	90.00	90.00 [9]	90.00 [11]	120.00	120.00	120.00 [12]	120.01 [13]

The structure of a given amorphous oxide (e.g. its density) strongly depends on the synthesis method (both in experiments and atomistic modeling), which renders it unsuitable for benchmarking the model. Therefore, our analysis shifts to the disordered structure of liquid alumina, as modeled using both PFP and PFP+D3, against experimental data derived from neutron scattering [14] and X-ray scattering [15] experiments. The comparative results are illustrated in Fig. S1, which presents an overlay of the radial distribution functions $g(r)$ for stoichiometric liquid alumina. As shown in Fig. S1, the comparison between the liquid alumina structures modeled by PFP and those by PFP+D3 reveals negligible differences, while the predicted liquid density affirms the applicability of the PFP+D3 potential for studying alumina and for subsequent analyses within this study.

Noteworthy, the mean Al-O bond length across all methodologies hovers around $r \approx 1.76$ Å, indicating a consistent representation of this fundamental structural parameter. However, discrepancies arise when considering the position of the second peak at ≈ 2.9 Å, which corresponds to both O-O and Al-Al distances [16]. Typically, neutron scattering provides more accurate information for lighter elements like oxygen, whereas X-ray scattering is more effective for electron-rich atoms such as aluminum. Consequently, the overlap in the second peak leads to a broader experimental peak, whereas atomistic simulations display a more defined second peak position that does not fully match either experimental measurement. This misalignment suggests potential limitations in the experimental resolution or the simulation model's ability to concurrently capture the dynamics of both light and heavy elements with equal precision.

The structural composition of liquid alumina, as evidenced through neutron scattering [14], [16], NMR [17], and X-ray studies [15], represents a macroscopic averaging of various local atomic arrangements. These arrangements, rather than being static, are part of a dynamic equilibrium, contributing to the complexity and connectivity observed within the liquid alumina structure. However, it is essential to recognize that the coordination numbers derived from neutron scattering data, which suggest a lower density for the high-temperature liquid compared to sapphire [16], reflect the alterations in aluminum and oxygen coordination in response to temperature variations.

This diverse spectrum of experimental evidence highlights the challenge of pinpointing a definitive structural model for liquid alumina. The dynamic nature of atomic coordination and the variability across different experimental methods suggest that the true structure likely embodies

aspects of each observed configuration. In this context, the PFP potential emerges as a promising tool for bridging these experimental observations, offering a comprehensive model that may well align with the multifaceted reality of liquid alumina's structure.

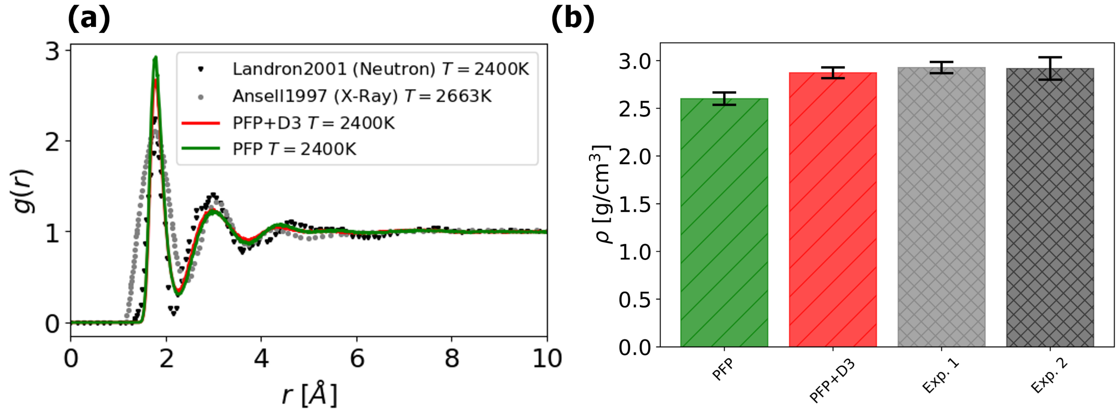

Figure S1: (a) Total radial distribution functions, $g(r)$, for liquid alumina at a temperature of $T = 2400$ K. The presented data are compared against experimental benchmarks derived from neutron scattering and x-ray experiments conducted by Landron *et al.* [14], and Ansell *et al.* [15], respectively. (b) The density of liquid alumina for PFP and PFP+D3 was computed from 100K steps of an NPT equilibration run that preceded the NVT run used to calculate $g(r)$. The experimental reference data is included for comparison. Exp. 1 corresponds to the electrostatic levitation method [18] while Exp. 2 corresponds to the neutron diffraction method [19].

We have also quantitatively validated our model by comparing energies and forces against DFT for all systems investigated, as shown in Fig. S2. The DFT data employed for this validation consists of the ionic relaxations from the final frame of the PFP+D3 calculations. These relaxations were used to derive the structures for the Bader charge analysis, as described in Sec. 4.3 of the main text. Notably, the energy errors—RMSE of 16.856 meV and MAE of 16.341 meV—and the force errors—RMSE of 81.175 meV/Åand MAE of 44.108 meV/Å—are lower than those reported for several other universal potentials (MACE(RMSE(energy) = 74 meV/atom), M3GNet(RMSE(energy) = 34 meV/atom), CHGNet(RMSE(energy) = 54 meV/atom)) [20] and fall within the range observed for machine learning potentials targeted to multicomponent oxide glasses (RMSE(energy) = 26 meV/atom, RMSE(force)=203 meV/Å)[21], or specifically trained on amorphous alumina with high density and stoichiometric variation (RMSE(energy)=16 meV/atom, RMSE(force) = 203meV/Å)[22]. Moreover, the computed formation energies, E_{f}, are in close agreement with the DFT values, although with a systematic offset, as illustrated in Fig. S3. This offset can be minimized by adjusting the reference DFT settings i.e., by tuning the number of k-points or D3 correction formalism, as demonstrated for the Bayerite case (see Fig. S3). As we do not know exactly which DFT settings have been used for computing relevant training datasets by PFP developers, we believe that our results indicate the exceptional ability of PFP in atomistic modeling amorphous alumina with hydrogen incorporation at a state-of-the-art level of accuracy.

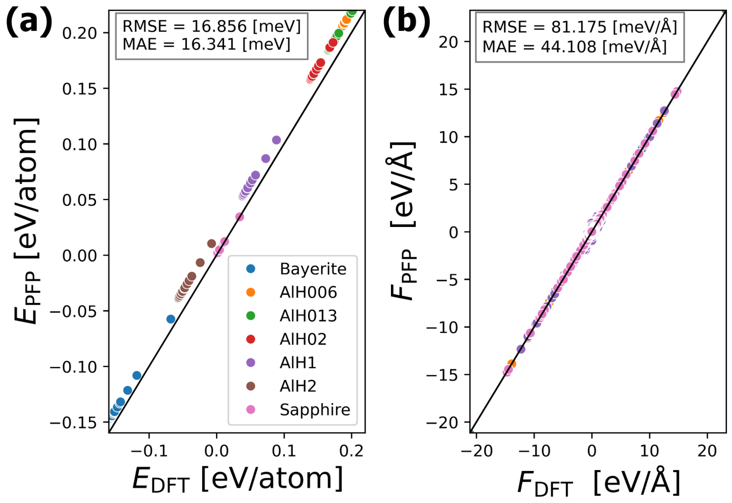

Figure S2: (a) The accuracy of the PFP+D3 potential in terms of the formation energy. As a reference for formation energy calculation, the energy per Al_2O_3 formula unit in α-Al_2O_3 and the energy of a single water molecule in vacuum were employed. (b) The accuracy of the PFP+D3 potential for force prediction. The plot compiles the x, y, and z components of force vectors.

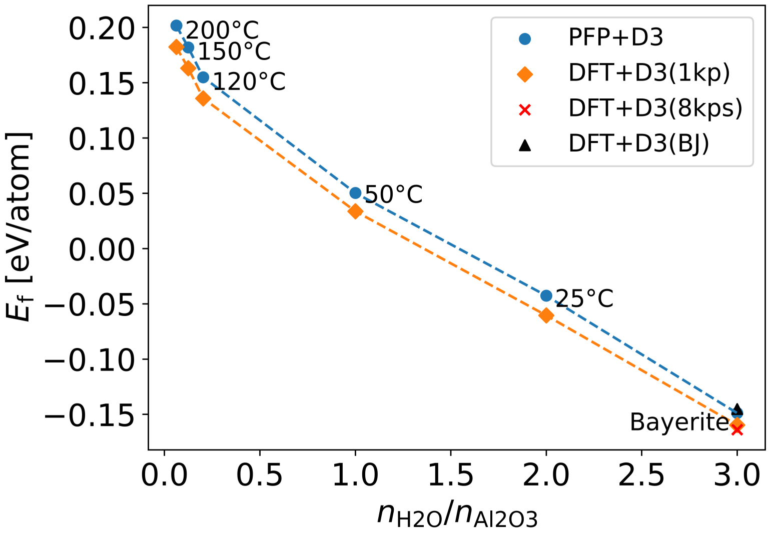

Figure S3: Formation energy per atom, E_f, of all investigated structures as a function of the water-to-alumina ratio, n_{H2O}/n_{Al2O3}. The energies of all compounds are computed using the DFT+D3 with one k-point and zero damping. The open circles denote the DFT+D3-baseline data, while DFT+D3 relaxations for Bayerite obtained using 8 k-points (red cross) and those using the Becke–Johnson D3 correction (black triangle) are shown. This plot illustrates how the selection of the DFT settings affects the systematic offset in the energy accuracy of the PFP+D3 potential.

S2 Atomistic Simulation Cells

Table S2: Details of the simulations cells used in the atomistic simulations including type of phase, ALD growth temperature, H/Al ratio, numbers of Al, O, H atoms, and cell parameters a, b, c, α, β, γ.

Sample	H/Al ratio	# Al	# O	# H	a [Å]	b [Å]	c [Å]	α [°]	β [°]	γ [°]
Sapphire	0.00	108	162	0	14.39	14.39	13.08	90.00	90.00	120.00
Bayerite	3.00	128	384	384	20.33	17.46	18.68	90.00	90.41	90.00
Amorphous (25°C)	2.00	128	320	256	19.97	16.74	18.60	90.18	91.03	90.38
Amorphous (50°C)	1.00	128	256	128	19.14	17.04	14.49	90.29	90.43	88.13
Amorphous (120°C)	0.20	128	205	26	16.89	14.45	15.53	89.21	89.78	88.55
Amorphous (150°C)	0.13	128	200	16	16.74	14.65	15.05	92.33	88.29	88.46
Amorphous (200°C)	0.06	128	196	8	16.58	14.00	15.26	89.90	92.28	88.93

S3 Geometrical factor, D, and Al-O bond length, r, in relation to coordination number, n

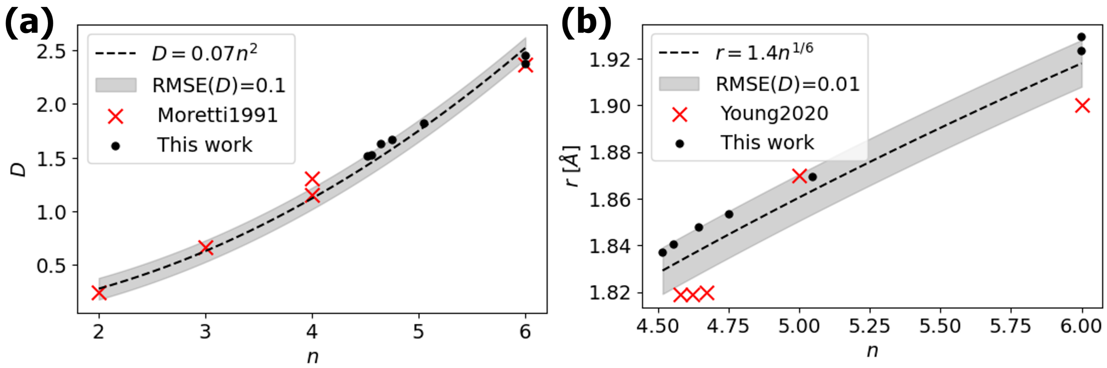

Figure S4: (a) Dependency of the geometrical factor, D, on the coordination number, n. The data is fitted with a power law, $D = D_0 n^2$, using the least squares error method, where $D_0 = 0.07$. Structures used in this study are shown in black, and those from [23] are shown in red. The grey area around the fitted curve represents the root mean square error (RMSE) of 0.1. (b) Dependency of the bond length, r, on the coordination number, n. The data is fitted with a power law, $r = r_0 n^{1/6}$, using the least squares error method, where $r_0 = 1.4$. Structures used in this study are shown in black, and those from [24] are shown in red. The grey area around the fitted curve represents the RMSE of 0.01.

S4 O charge isosurfaces for sapphire and bayerite

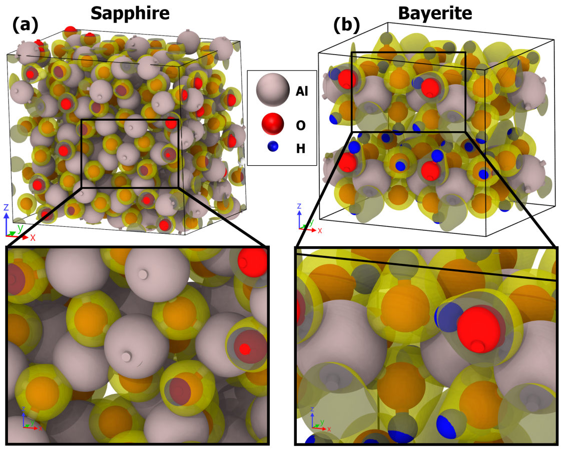

Figure S5: Isosurfaces in the crystalline reference phases (a) Sapphire and (b) Bayerite, as derived from *ab initio* calculations (see Methods). The isosurface level used is 0.5 e/Å^3.

References

[1] D. L. Duong, S. J. Yun, and Y. H. Lee, "Van der Waals Layered Materials: Opportunities and Challenges," *ACS Nano*, vol. 11, no. 12, pp. 11 803–11 830, 2017, ISSN: 1936086X. DOI: 10.1021/acsnano.7b07436.

[2] A. Lozano, B. Escribano, E. Akhmatskaya, and J. Carrasco, "Assessment of van der Waals inclusive density functional theory methods for layered electroactive materials," *Phys. Chem. Chem. Phys.*, vol. 19, no. 15, pp. 10 133–10 139, 2017, ISSN: 14639076. DOI: 10.1039/c7cp00284j.

[3] J. Moellmann and S. Grimme, "DFT-D3 study of some molecular crystals," *J. Phys. Chem. C*, vol. 118, no. 14, pp. 7615–7621, 2014, ISSN: 19327455. DOI: 10.1021/jp501237c.

[4] G. Graziano, J. Klimeš, F. Fernandez-Alonso, and A. Michaelides, "Improved description of soft layered materials with van der Waals density functional theory," *J. Phys. Condens. Matter*, vol. 24, no. 42, 2012, ISSN: 09538984. DOI: 10.1088/0953-8984/24/42/424216.

[5] S. Grimme, J. Antony, S. Ehrlich, and H. Krieg, "A consistent and accurate ab initio parametrization of density functional dispersion correction (DFT-D) for the 94 elements H-Pu," *J. Chem. Phys.*, vol. 132, no. 15, 2010, ISSN: 00219606. DOI: 10.1063/1.3382344.

[6] S. Grimme, S. Ehrlich, and L. Goerigk, "Effect of the damping function in dispersion corrected density functional theory," *J. Comput. Chem.*, vol. 32, no. 7, pp. 1456–1465, 2011, ISSN: 0192-8651. DOI: 10.1002/jcc.21759. [Online]. Available: https://onlinelibrary.wiley.com/doi/10.1002/jcc.21759.

[7] J. Hermann, R. A. DiStasio, and A. Tkatchenko, "First-Principles Models for van der Waals Interactions in Molecules and Materials: Concepts, Theory, and Applications," *Chem. Rev.*, vol. 117, no. 6, pp. 4714–4758, 2017, ISSN: 15206890. DOI: 10.1021/acs.chemrev.6b00446.

[8] Pfnet-research, *Torch-dftd*, 2023. [Online]. Available: https://github.com/pfnet-research/torch-dftd.

[9] J. D. Gale, A. L. Rohl, V. Milman, and M. C. Warren, "An ab initio study of the structure and properties of aluminum hydroxide: Gibbsite and bayerite," *J. Phys. Chem. B*, vol. 105, no. 42, pp. 10 236–10 242, 2001, ISSN: 10895647. DOI: 10.1021/jp011795e.

[10] C. Cancellieri, S. Gramatte, O. Politano, *et al.*, "Effect of hydrogen on the chemical state, stoichiometry and density of amorphous Al2O3 films grown by thermal atomic layer deposition," *Surf. Interface Anal.*, vol. 56, no. 5, pp. 293–304, 2024, ISSN: 0142-2421. DOI: 10.1002/sia.7282. [Online]. Available: https://analyticalsciencejournals.onlinelibrary.wiley.com/doi/10.1002/sia.7282.

[11] F. Zigan, W. Joswig, and N. Burger, "Die Wasserstoffpositionen im Bayerit, Al(OH)3," *Zeitschrift für Krist. - Cryst. Mater.*, vol. 148, no. 3-4, pp. 255–274, 1978. DOI: doi:10.1524/zkri-1978-3-407. [Online]. Available: https://doi.org/10.1524/zkri-1978-3-407%20https://www.osti.gov/biblio/1187823.

[12] T. M. Project, "Materials Data on Al2O3 by Materials Project," *Mater. Proj.*, 2020. DOI: 10.17188/1187823. [Online]. Available: https://www.osti.gov/biblio/1187823.

[13] L. W. Finger and R. M. Hazen, "Crystal structure and compression of ruby to 46 kbar," *J. Appl. Phys.*, vol. 49, no. 12, pp. 5823–5826, 1978, ISSN: 00218979. DOI: 10.1063/1.324598.

[14] C. Landron, A. K. Soper, T. E. Jenkins, G. N. Greaves, L. Hennet, and J. P. Coutures, "Measuring neutron scattering structure factor for liquid alumina and analysing the radial distribution function by empirical potential structural refinement," *J. Non. Cryst. Solids*, vol. 293-295, no. 1, pp. 453–457, 2001, ISSN: 00223093. DOI: 10.1016/S0022-3093(01)00839-0.

[15] S. Ansell, S. Krishnan, J. K. R. Weber, *et al.*, "Structure of Liquid Aluminum Oxide," *Phys. Rev. Lett.*, vol. 78, no. 3, pp. 464–466, 1997, ISSN: 0031-9007. DOI: 10.1103/PhysRevLett.78.464. [Online]. Available: https://link.aps.org/doi/10.1103/PhysRevLett.78.464.

[16] C. Landron, L. Hennet, T. E. Jenkins, G. N. Greaves, J. P. Coutures, and A. K. Soper, "Liquid alumina: Detailed atomic coordination determined from neutron diffraction data using empirical potential structure refinement," *Phys. Rev. Lett.*, vol. 86, no. 21, pp. 4839–4842, 2001, ISSN: 00319007. DOI: 10.1103/PhysRevLett.86.4839.

[17] P. Florian, D. Massiot, B. Poe, I. Farnan, and J. P. Coutures, "A time resolved 27Al NMR study of the cooling process of liquid alumina from 2450 °C to crystallisation," *Solid State Nucl. Magn. Reson.*, vol. 5, no. 3, pp. 233–238, 1995, ISSN: 09262040. DOI: 10.1016/0926-2040(95)01188-X.

[18] P.-F. Paradis, T. Ishikawa, Y. Saita, and S. Yoda, "Non-Contact Thermophysical Property Measurements of Liquid and Undercooled Alumina," *Jpn. J. Appl. Phys.*, vol. 43, no. 4R, p. 1496, 2004, ISSN: 0021-4922. DOI: 10.1143/JJAP.43.1496. [Online]. Available: https://iopscience.iop.org/article/10.1143/JJAP.43.1496.

[19] L. B. Skinner, A. C. Barnes, P. S. Salmon, *et al.*, "Joint diffraction and modeling approach to the structure of liquid alumina," *Phys. Rev. B*, vol. 87, no. 2, p. 024 201, Jan. 2013, ISSN: 1098-0121. DOI: 10.1103/PhysRevB.87.024201. [Online]. Available: https://link.aps.org/doi/10.1103/PhysRevB.87.024201.

[20] R. Jacobs, D. Morgan, S. Attarian, *et al.*, "A practical guide to machine learning interatomic potentials – Status and future," *Curr. Opin. Solid State Mater. Sci.*, vol. 35, p. 101 214, Mar. 2025, ISSN: 13590286. DOI: 10.1016/j.cossms.2025.101214. [Online]. Available: https://linkinghub.elsevier.com/retrieve/pii/S1359028625000014.

[21] R. Kayano, Y. Inagaki, R. Matsubara, K. Ishida, and T. Ohkubo, "Development and Validation of Neural Network Potentials for Multicomponent Oxide Glasses," *J. Phys. Chem. C*, vol. 128, no. 41, pp. 17 686–17 702, Oct. 2024, ISSN: 1932-7447. DOI: 10.1021/acs.jpcc.4c04604. [Online]. Available: https://pubs.acs.org/doi/10.1021/acs.jpcc.4c04604.

[22] W. Li, Y. Ando, and S. Watanabe, "Effects of density and composition on the properties of amorphous alumina: A high-dimensional neural network potential study," *J. Chem. Phys.*, vol. 153, no. 16, 2020, ISSN: 10897690. DOI: 10.1063/5.0026289. [Online]. Available: https://doi.org/10.1063/5.0026289.

[23] G. Moretti, "Auger parameter shifts in the case of the non-local screening mechanism: Applications of the electrostatic model to molecules, solids and adsorbed species," *Surf. Interface Anal.*, vol. 17, no. 6, pp. 352–356, 1991, ISSN: 10969918. DOI: 10.1002/sia.740170610.

[24] M. J. Young, N. M. Bedford, A. Yanguas-Gil, *et al.*, "Probing the Atomic-Scale Structure of Amorphous Aluminum Oxide Grown by Atomic Layer Deposition," *ACS Appl. Mater. Interfaces*, vol. 12, no. 20, pp. 22 804–22 814, 2020, ISSN: 1944-8244. DOI: 10.1021/acsami.0c01905. [Online]. Available: https://pubs.acs.org/doi/10.1021/acsami.0c01905.
